# Students’ associations with the STEM acronym and their impact on value beliefs and STEM choices

**DOI:** 10.1111/nyas.70018

**Published:** 2025-08-25

**Authors:** Heidrun Stoeger, Anton L. Beer, Albert Ziegler

**Affiliations:** ^1^ Fakultät für Humanwissenschaften Universität Regensburg Regensburg Germany; ^2^ Institut für Psychologie Friedrich‐Alexander‐Universität Erlangen‐Nürnberg Nürnberg Germany

**Keywords:** academic elective intentions, school career choices, school subjects, STEM, value beliefs

## Abstract

In recent decades, there have been many campaigns to attract students to STEM study programs and jobs. However, there is little research on whether the target audiences are familiar with the STEM acronym, which specific STEM subject areas they associate with it, and the impact of these associations. We investigated students’ familiarity with the STEM acronym and whether their associations of the STEM acronym with different STEM subject areas—mediated by their value beliefs—affected their academic elective intentions for STEM study programs and activities and their STEM choices of curriculum profiles at school. In a sample of eighth‐grade students (*n* = 1163; 611 girls; 13.7 years), 72% reported familiarity with the STEM acronym. Students associated mathematics most strongly with the STEM acronym, followed by physics, computer science, chemistry, biology, and engineering. The subject areas students associate with the STEM acronym affected their academic elective intentions for STEM and their STEM choices at school. These relations were mediated by students’ value beliefs and differed for the subject areas associated with the STEM acronym and by gender. The consequences of our findings for tailoring STEM campaigns to ensure their effectiveness and a more diverse and inclusive STEM community are discussed.

## Graphical abstract

The perceived meaning of the acronym STEM (science, technology, engineering, mathematics) affected value beliefs and school career decisions for STEM. Associations of the STEM acronym with mathematics increased cost beliefs, which in turn decreased decisions for STEM careers. Associations with physics and computer science increased valence beliefs in boys, and associations with biology increased valence beliefs in girls, which in turn increased decisions for STEM careers.

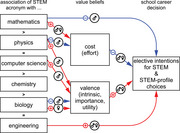

## INTRODUCTION

In many countries, elective intentions for STEM (science, technology, engineering, and mathematics) and STEM choices are rather low. This is already evident during the school years.^1^, ^2^ A similar picture emerges for STEM degree program choices. While the share of new tertiary graduates in STEM in 2021 was 41% in China^3^ and 37% in Russia,^4^ the highest share for a country of the Organisation for Economic Co‐operation and Development (OECD) was 35% in Germany.^5^ In the United States, it was only 24%, and in the OECD country with the lowest share, Norway, only 16%. As a result, there is a skills gap in STEM in many countries. For example, Germany recorded a STEM skills gap of 285,800 in 2023.^6^ The STEM skills gap in the United States is estimated at around one million.^7^, ^8^

In response to this challenging situation, attempts have been made for decades to attract more individuals to STEM fields with the help of media and political advertising campaigns.^9^, ^10^, ^11^ In many cases, STEM courses, degree programs, and professions are widely advertised under the STEM acronym. Similar acronyms are used in numerous languages including French (STIM: science, technologie, ingénierie et mathématiques), German (MINT: Mathematik, Informatik, Naturwissenschaft und Technik), Italian (STIM: scienza, tecnologia, ingegneria e matematica), Spanish (CTIM: ciencia, tecnología, ingeniería y matemáticas), Turkish (FeTeMM: fen, teknoloji, mühendislik ve matematik), Urdu (انجینئرنگ, ریاضی, اِسٹیم: سائنس, ٹیکنالوجی,), and Mandarin (理工科: 科学、技术、工程、数学). Indeed, many well‐known current educational programs or associations have the term STEM directly in their name, for example: “NASA STEM Engagement”,^12^ “STEM Nova Awards”,^13^ “STEM Connector”,^14^ “STEM Education Coalition”,^15^ or “STEM Next Opportunity Fund”.^16^ Other programs use the acronym when addressing the public.^17^, ^18^, ^19^, ^20^ However, using the STEM acronym poses various problems. For example, it is unclear whether the relevant groups to be addressed know the STEM acronym and with which subject areas it is associated. A more precise understanding of these associations seems essential for at least two reasons.

First, the STEM sector is highly differentiated. The absolute participation rates for the individual STEM areas differ significantly. For example, the skills gap in the United States is very pronounced for some subject areas, such as architecture and engineering. In contrast, there is even a surplus of qualified professionals in other areas, such as computer and mathematics‐related fields.^21^ In addition, subgroup‐specific choices can be observed in individual STEM subject areas. One subgroup that receives much attention in research is women.^22^ Women are less likely to choose mathematics‐intense STEM subject areas such as mathematics and statistics, computer science, engineering, or physical and technical sciences. At the same time, they are overrepresented in other STEM subject areas, such as biological and biomedical sciences.^23^

Second, the reasons for the low elective intentions for STEM and STEM choices are closely linked to the perception of the STEM field as a whole, but in particular to a different perception of the individual STEM subject areas. For example, value beliefs for the individual STEM subject areas sometimes differ significantly.^24^, ^25^, ^26^ Since value beliefs are a significant predictor of elective intentions for STEM and STEM choices,^27^, ^28^ it can be assumed that associations of the STEM acronym with different STEM subject areas, mediated by value beliefs, could influence elective intentions for STEM and STEM choices. Our study aims to test this assumption empirically.

### The STEM acronym: Familiarity and associations

There is little research on whether individuals are familiar with the STEM acronym and, if so, with which subject areas they associate it. The few existing studies suggest that not all individuals are familiar with the STEM acronym. For instance, Breiner et al.^29^ found that in a survey of college faculty members at a research institution, 73% were familiar with the STEM acronym. However, 9% of those who indicated that they knew the acronym incorrectly stated that the “M” stands for medicine. Similarly, Adler et al.^30^ found that 85% of college students correctly associated the letters of the STEM acronym with the respective STEM subject areas, but 4% were not familiar with it at all, 4% could not correctly identify at least one of the letters, and 2% each incorrectly stated that “M” stands for medicine instead of mathematics and “E” stands for electrical/electric instead of engineering. Only among students majoring in STEM were all able to identify the acronym correctly. A survey by the Entertainment Industries Council found that 86% of the 5000 participants did not understand the STEM acronym, with some stating that it stands for STEM cell research or flower, or broccoli stem.^31^ A study by the MINTality Foundation^32^ asked 1500 Austrian secondary school students what the acronym STEM (in German: MINT) means. Only 30% were familiar with the German equivalent of the STEM acronym.

These results indicate that the STEM acronym is predominantly unknown to people with little exposure to STEM and that even those familiar with the STEM acronym sometimes associate the wrong STEM subject areas with the letters it contains. This appears problematic for advertising campaigns for STEM learning opportunities, degree programs, and professions, as such efforts are likely ineffective or less effective with people unfamiliar with the STEM acronym. Moreover, some initial hints suggest that even among individuals familiar with the STEM acronym, campaigns may have different effects and will elicit different decisions for or against STEM depending on the subject areas with which the individuals associate the STEM acronym. For instance, Adler et al.^30^ showed that the values and self‐reported interest in a STEM career of 10th graders in the United States differed depending on whether they assumed that the STEM acronym also includes medicine. Self‐reported interest in a STEM career was 42% if medicine was included, but only 13% if not. These results suggest that associations of the STEM acronym with different STEM subject areas might influence STEM‐related elective intentions and choices. According to the situated expectancy–value theory,^27^, ^28^ this relationship is likely mediated by value beliefs.

### Value beliefs and their significance for STEM‐related elective intentions and choices

Situated expectancy–value theory^27^, ^28^, ^33^ postulates various causes for task and activity choices in STEM, which operate at different levels and interact in multiple ways. In addition to macro‐ and distal aspects at the socio‐historical‐cultural level (e.g., stereotypes about abilities and the nature of abilities), more proximal but still longer‐term socialization experiences (e.g., attitudes and behaviors of parents or teachers) as well as more direct cognitive processes (e.g., the self‐concept of one's abilities or goals) affect task and activity choices. Individual expectations of success and subjective task values (i.e., value beliefs) are the most proximal psychological determinants of task and activity choices. Expectations of success and task values are influenced in various ways by the distal causes.

Individuals choose to engage in tasks, activities, and domains in which they expect to succeed and that have value for them. Research findings indicate that value beliefs predict elective intentions and choices and do so more strongly than competence‐related beliefs.^1^, ^34^ For example, Jiang et al.^35^ found that adolescents’ math and science value beliefs at the beginning of high school were positively associated with STEM achievement and course choices throughout high school and college major choices 7 years later.

Value beliefs comprise various facets such as intrinsic value, attainment value, utility value, and cost,^28^, ^36^ which are sometimes classified into a valence component and a cost component.^25^, ^37^ Particularly when considering value beliefs for different STEM subject areas, these two components should be considered separately.^27^ For example, Vinni‐Laakso et al.^24^ found differences in valence and cost assessments for mathematics, biology, and physics in Finnish seventh‐ to ninth‐grade students. While valence was rated highest for mathematics, followed by biology and then physics, cost was rated highest for mathematics, followed by physics and then biology. Gaspard et al.^26^ also found differences in valence and cost ratings for different STEM subject areas in German eighth‐ to 12th‐grade students. Gender differences were also evident. The valence of biology was rated higher by girls than boys, while boys rated the valence of physics higher than girls. Boys rated the cost of biology and mathematics higher than girls, while girls rated the cost of physics higher than boys.

Only a few studies investigated valence and cost assessments for multiple STEM subject areas. In two studies, value beliefs for languages were surveyed in addition to value beliefs for three STEM subject areas (mathematics, biology, and physics). Other studies have either focused solely on the valence facet^38^, ^39^ or assessed value beliefs for science without distinguishing individual subject areas.^40^, ^41^ There is also a research gap on whether associations of the STEM acronym with different STEM subject areas relate to different valence and cost beliefs for STEM. Should this be the case, associations of the STEM acronym with different STEM subject areas—mediated by value beliefs (and, here in particular, valence and cost)—could be expected to influence elective intentions and choices.

### Current study

To assess the impact of broad STEM promotional campaigns and offerings to increase STEM participation, it is essential to understand whether individuals are familiar with the STEM acronym and with which subject areas they most strongly associate the STEM acronym. The latter seems to be important as value beliefs differ for different STEM subject areas,^30^, ^38^, ^39^ and value beliefs are proximal predictors of STEM elective intentions and choices.^27^, ^28^ It can, therefore, be assumed that associations of the STEM acronym with different STEM subject areas, mediated by value beliefs, are related to STEM elective intentions and choices.

Our study investigated this question among eighth‐grade students, who had to make a profile choice (study focus on STEM or non‐STEM). In the German school system,^42^ secondary school students choose a specific academic focus, a profile. They can select from several options, including STEM (Science, Technology, Engineering, and Mathematics), language, music, and others, such as art. This choice of profile is not based on achievement levels. The selected profile influences the curriculum and determines the number of hours per week students spend on subjects related to their focus. For instance, students who opt for the STEM profile receive additional instruction in mathematics and science compared to those who choose other profiles. The decision about which profile to pursue is made at different grade levels depending on the federal state. In the states we examined, students typically make this choice in the eighth grade.

Our first aim was to investigate whether students were familiar with the STEM acronym and which STEM subject areas they most strongly associated with the STEM acronym. A further aim of our study was to gain a more detailed understanding of the significance of associations of the STEM acronym with different STEM subject areas. In particular, we investigated whether associations of the STEM acronym with different STEM subject areas relate to academic elective intentions for STEM and STEM‐profile choices. The study by Adler et al.^30^ suggests that associations of the STEM acronym with different STEM subject areas are related to value beliefs, and numerous studies show that value beliefs are related to elective intentions and choices.^27^, ^28^ Based on these findings, we assumed that and investigated whether associations with the STEM acronym with different STEM subject areas—mediated by value beliefs—are related to elective intentions for STEM and STEM profile choices. We examined these associations for the total sample and girls and boys separately. Specifically, we investigated the following six research questions:

1. Are students familiar with the STEM acronym?
2. Which subject areas do students most strongly associate with the STEM acronym?
3. Are associations of subject areas with the STEM acronym related to academic elective intentions and STEM‐profile choices?
4. Are value beliefs in STEM (valence and cost) related to academic elective intentions for STEM and STEM‐profile choices?
5. Are students’ associations of subject areas with the STEM acronym related to value beliefs (valence and cost) for STEM?
6. Are students’ associations of subject areas with the STEM acronym and their relations to elective intentions for STEM and STEM‐profile choices mediated by value beliefs?

## METHODS

### Sample

The online survey was conducted at 26 German grammar schools (drawn from eight German states). Participation of schools and students was voluntary. This study was approved by the ministries of participating German states and the principals of participating schools. Only students who gave informed assent and whose parents gave informed consent participated in the survey. The survey was part of a larger 5‐year longitudinal research project with two cohorts that started either in the fall of 2022 or 2023, respectively. Only data from the first measurement point and students in the eighth grade are reported here. As the superordinate project is on STEM education, all participating schools offered special STEM activities (e.g., Junior‐Ingenieur‐Akademie, www.telekom‐stiftung.de/aktivitaeten/junior‐ingenieur‐akademie). Moreover, most schools were from larger cities (66% of the students from cities with more than a million, and another 15% from cities with more than 100,000 residents). Among the participating schools and classes, 45% of the students (participation rate) responded to the survey. The percentage of girls among respondents (53%) was slightly higher than the percentage of girls in participating classes (51%), but this difference was not significant (*p* = 0.20) as tested by a chi‐square test of the contingency table.

Overall, 1163 students participated in the survey. However, 91 datasets had to be excluded because four students were outside the age range (12−16 years), 69 students did not respond to the main questions or showed suspicious response patterns (i.e., straightlining in multi‐item scales even for reverse‐coded items) or did not identify as male or female (18 students). Of the remaining 1072 students, 611 were girls (mean age: 13.7 years, range: 12.1−15.8), and 461 were boys (mean age: 13.7 years, range: 12.1−15.3). For 60% of the students, at least one parent had a college‐level education, and 10% were born outside of Germany. See details of the demographics in Table S1.

### Procedure

The survey was administered in German online using LimeSurvey (Limesurvey GmbH, www.limesurvey.org). Only students who received an individual access code could participate. Access codes were distributed to students by their teachers contingent on their consent. Students could interrupt or quit the survey at any time. After successful submission, the individual access code became invalid. As the survey was part of a larger research project, it also included questions not relevant to the purpose of this study. Overall, the survey took about 40 min to complete.

### Measures

#### Familiarity with the STEM acronym

At the beginning of the survey, students were informed that STEM (in German: MINT) is an acronym used to refer to the academic disciplines of Science, Technology, Engineering, and Mathematics. As the survey was administered in German, the explanation consisted of the German equivalents: Mathematik, Informatik, Naturwissenschaften (Biologie, Physik, Chemie), Technik (in English: mathematics, computer science, natural science [biology, physics, chemistry], engineering). Students were asked to indicate (with *yes* or *no*) whether they knew what this acronym stands for before reading the explanation.

#### Association of subject areas with the STEM acronym

To measure students’ associations of subject areas with the STEM acronym, they were asked the following question: “Which subject area do you associate most with STEM?”. This question was administered with the LimeSurvey question type “Ranking.” Below the question, six boxes containing subject areas (mathematics, physics, computer science, biology, chemistry, and engineering) were presented in randomized order. Students were asked to rank the subject areas by dragging the boxes into a new field at the right, whereby the subject area students associated strongest with the STEM acronym was to be listed on top, and the subject area they associated least with the STEM acronym was to be listed at the bottom of the field. The responses were scored per subject area according to the position in the response list from 1 (*least associated with the STEM acronym*) (i.e., at the bottom of the list) to 6 (*most associated with the STEM acronym*) (i.e., at the top of the list). Note that owing to the assessment procedure, the subject area associations with the STEM acronym were partially statistically dependent (multicollinear).

#### Value beliefs for STEM

We measured students’ value beliefs for STEM with 10 items by adapting a scale by Gaspard and colleagues^25^ and Jiang and colleagues.^43^ In order to probe several facets of value beliefs, eight items from the subscales (two from each), “intrinsic,” “personal importance,” “general utility (for future life),” and “effort required” were selected from Gaspard and colleagues.^25^ These items were supplemented by two items from the “cost” subscale of Jiang and colleagues.^43^ The items (see also Table S2) included questions such as: “I simply like STEM subjects.” for the intrinsic facet; “It is important to me to know a lot in STEM subjects.” for the personal importance facet; “The contents of STEM subjects will help me in my life.” for the general utility (for future life) facet; “Dealing with STEM subjects is exhausting to me.” for the effort required facet; and “It takes too much of effort for me to do well in STEM subjects.” for the cost facet. All items were rated on a 6‐point Likert scale ranging from 1 (*completely disagree*) to 6 (*completely agree*).

Although the scale aimed to probe five facets of value beliefs, our scale evaluation showed a hierarchical factor structure. A principal axes factor analysis with five factors (with oblique promax rotation) showed that the five factors were highly correlated around two relatively independent factors. A factor analysis with two factors (with oblique promax rotation) showed that the items probing the intrinsic, personal importance, general utility (for life) facets highly loaded (all loadings ≥ 0.658) on one factor (with poor loadings of ≤ 0.300 on the second factor), and the items probing the effort required and cost facets highly loaded (≥ 0.742) on the second factor (with poor loadings of ≤ 0.080 on the first factor) with a relatively low interfactor correlation (*r* = −0.344) (see also Table S2). Accordingly, the scale was split into two parts (subscales). The first subscale comprised six items, which reflected the intrinsic, personal importance, and general utility (for life) facets of Gaspard and colleagues.^25^ As these facets reflect positive values, we labeled this subscale as “valence” for this study. The second subscale comprised the remaining four items that reflected the effort required facet of Gaspard and colleagues^25^ and the cost facet of Jiang and colleagues.^43^ We labeled this subscale “cost” for this study. The reliability of the combined scale was omega total = 0.917. Cronbach's alpha was α = 0.892 for the valence subscale and α = 0.875 for the cost subscale.

#### Academic elective intentions for STEM

Students’ academic elective intentions for STEM were measured with six items adapted from a scale by Stoeger and colleagues.^44^ The six items examined elective intentions concerning different STEM‐related activities. Sample items are “I can picture myself pursuing a career in the STEM domain.” and “I can picture myself participating in activities such as STEM competitions or Olympics.” All items (see also Table S3) were rated on a 6‐point Likert scale ranging from 1 (*completely disagree*) to 6 (*completely agree*). Cronbach's alpha for the total scale was α = 0.889.

#### STEM‐profile choices at school

In the German states where we conducted the study, students in secondary school were asked to make a decision about a particular academic focus (called a profile) at school. They can choose between several profiles: a STEM profile, a language profile, a musical profile, and others (e.g., art). This choice of profile is not based on achievement levels. The profiles affect the curriculum and determine how much instruction (lessons per week) students will receive in the subjects of the focus discipline. For example, students who select the STEM profile receive additional mathematics and science instruction compared to students who select other profiles. Profile choices occur at different grade levels, depending on the school and state.^42^ In our questionnaire, students indicated whether they plan to choose a STEM profile at their school in the upcoming school year. The question was, “Will you have to choose between profiles at your school in the upcoming year?”. Students responded with *yes* or *no*. If *yes* was selected, students received the question “Which profile(s) will you choose?”. If *no* was selected, the question was rephrased as “If you were to choose a profile at your school in the upcoming year, which profile would you choose?”. Responses were scored 1 if the STEM profile was selected and 0 if no STEM profile was selected.

### Analyses

All statistical analyses were performed in R^45^ using the *lavaan*,^46^ *psych*, *rms*, *rstatix*, and *stats* packages. Reliability assessments were performed using the alpha and omega functions. A chi‐square test for testing differences in familiarity with the STEM acronym was conducted with the *chisq.test* function. Analyses of variance (ANOVA), *t*‐tests, effect‐size estimates, and tests for equal variance were conducted by the *anova_test*, *t.test*, *cohens_d*, and *levene_test* functions, respectively. Corrections for unequal variances were applied when violations of equal variance were detected by Levene tests. Huynh−Feldt corrections were applied when a Mauchly test detected a violation of the sphericity assumption with an epsilon > 0.75. Correlational analyses were performed with the *corr.test* and *paired.r* (with Fisher z‐transformed correlation coefficients) functions, logistic regressions with the *lrm* function, and multiple regressions and the mediation analyses with the *sem* function. A maximum likelihood estimator was used for scaled variables, and a diagonally weighted least squares estimator with a mean and variance‐adjusted test statistic was used for categorical variables. The type‐I error was set to 0.05.

Note that our main analyses were primarily based on parametric procedures, although most of our data had an ordinal level of measurement (e.g., Likert scale responses). This was motivated by two reasons: first, parametric measures (e.g., mean) are more informative than nonparametric measures (e.g., median); second, the results of parametric tests are more comparable across analyses than those of nonparametric tests. Moreover, previous studies have shown that parametric procedures are acceptable even with Likert‐type data if composite scores or at least five response categories are used.^47^ This condition was met in our study. Nevertheless, we verified our analyses by additionally performing corresponding nonparametric tests (Friedman test, Wilcoxon test, Mann−Whitney test, and Spearman correlation test) using the R functions *friedman.test*, *wilcox.test*, and *corr.test*. As the results of these additional tests were very similar to those of the parametric tests, they are not reported.

## RESULTS

### Familiarity with the STEM acronym

Overall, 776 (72%) students indicated that they knew the STEM acronym. When girls and boys were considered separately, 485 (79%) of the girls and 291 (63%) of the boys knew the acronym. A chi‐square test of the contingency table showed that this ratio was significantly higher in girls than in boys (χ^2^(1, *N* = 1072) = 33.9, *p* < 0.001). For subsequent analyses, data from all students, irrespective of their response to the familiarity question, were analyzed. Control analyses with data containing only *yes* responses did not show substantially different result patterns (except for reduced power).

### Association of subject areas with the STEM acronym

**FIGURE 1:**
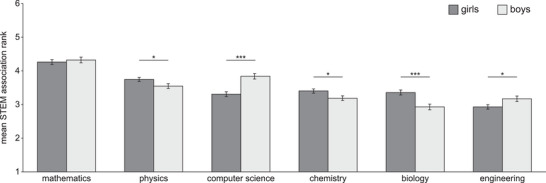
Association of subject areas with the STEM acronym. Bars show the mean associations of subject areas with the STEM acronym. Ranks were scaled from 1 (*least associated with*) to 6 (*most associated with*). Error bars show the standard error of the mean. The differences between girls (*n* = 639) and boys (*n* = 474) were compared by unpaired *t*‐tests. ^*^p ≤ 0.05; ^***^p ≤ 0.001.

We examined which subject areas were associated most/least with the STEM acronym. Students were asked to rank order the six subject areas. Associations were scaled from 1 to 6, whereby 6 reflects the subject area most associated with the STEM acronym. When girls and boys were analyzed together, mathematics was rated as most strongly associated with the STEM acronym, followed by physics, computer science, chemistry, biology, and engineering (Figure 1 and Table S4). A repeated‐measures ANOVA (Huynh−Feldt corrected) on the subject area association with the STEM acronym ranks showed a significant effect of the within‐subjects factor subject area (*F*(4.0, 4054.9) = 62.1, *p* < 0.001, 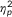 = 0.058). Subsequent paired‐sample *t*‐tests showed that mathematics was rated higher than physics (*t*(1014) = 8.0, *p* < 0.001, Cohen's *d* = 0.252), computer science was rated higher than chemistry (*t*(1014) = 2.5, *p* = 0.011, *d* = 0.079), and chemistry was rated higher than biology (*t*(1014) = 2.0, *p* = 0.041, *d* = 0.064). The comparisons of physics versus computer science (*p* = 0.13, *d* = 0.048) and biology versus engineering (*p* = 0.11, *d* = 0.050) were not significant.

When girls and boys were analyzed separately, associations with the STEM acronym differed across subject areas in both groups but with a different order. For girls, the ANOVA showed a significant effect of subject area (*F*(4.0, 2333.3) = 36.6, *p* < 0.001, 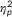 = 0.059). Paired‐sample *t*‐tests showed that mathematics was more associated with the STEM acronym than physics (*t*(581) = 5.0, *p* < 0.001, *d* = 0.206), physics was more associated than chemistry (*t*(581) = 4.2, *p* < 0.001, *d* = 0.175), and computer science was more associated than engineering (*t*(581) = 4.6, *p* < 0.001, *d* = 0.192). The comparisons of chemistry versus biology (*p* = 0.62, *d* = 0.021) and biology versus computer science (*p* = 0.68, *d* = 0.017) were not significant. For boys, the ANOVA also showed a significant effect of the subject area (*F*(4.1, 1759.5) = 35.5, *p* < 0.001, 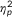 = 0.076). Paired‐sample *t*‐tests showed that mathematics was more associated with the STEM acronym than computer science (*t*(432) = 3.9, *p* < 0.001, *d* = 0.189), computer science more than physics (*t*(432) = 2.3, *p* = 0.020, *d* = 0.113), physics more than engineering (*t*(432) = 3.1, *p* = 0.002, *d* = 0.148), and chemistry more than biology (*t*(432) = 2.6, *p* = 0.009, *d* = 0.126). The engineering versus chemistry comparison was not significant (*p* = 0.89, *d* = 0.007).

A comparison of girls’ and boys’ associations by independent samples *t*‐tests showed that boys associated computer science (*t*(1013) = 4.9, *p* < 0.001, *d* = 0.313) and engineering (*t*(902) = 2.4, *p* = 0.019, *d* = 0.150) more strongly with the STEM acronym than girls, whereas girls associated physics (*t*(1013) = 2.1, *p* = 0.034, *d* = 0.135), chemistry (*t*(1013) = 2.2, *p* = 0.028, *d* = 0.140), and biology (*t*(1013) = 3.7, *p* < 0.001, *d* = 0.236) more strongly with the STEM acronym than boys. Gender differences in mathematics were not significant (*p* = 0.59, *d* = 0.034).

In summary, students associated mathematics most strongly with the STEM acronym, followed by physics, computer science, chemistry, biology, and engineering. Moreover, boys and girls differed in their association of subject areas with the STEM acronym. Boys associated computer science and engineering more with the STEM acronym than girls, whereas girls associated physics, chemistry, and biology more with the STEM acronym.

### Association of subject areas with the STEM acronym and their relation to academic elective intentions for STEM and STEM‐profile choices

Next, we wanted to know how students’ associations of subject areas with the STEM acronym related to academic elective intentions for STEM and STEM‐profile choices at school. As shown in Table 1, the association of subject areas with the STEM acronym was unrelated to academic elective intentions for STEM in the combined group (girls and boys). Simple regressions were calculated that predicted academic elective intention for STEM scores based on associations of subject areas with the STEM acronym scores. However, none of the association ranks of the six school subjects predicted academic elective intentions for STEM (all *p* ≥ 0.15). When groups were considered separately, no significant correlations or regression coefficients (all *p* ≥ 0.18) were observed for girls (Table 1). However, for boys, there were two positive and two negative correlations between the associations of school subjects with the STEM acronym and the academic elective intention for STEM (Table 1). Simple regressions showed that academic elective intentions for STEM were significantly predicted by associations of mathematics (*b* = −0.087, *t*(431) = −2.8, *p* = 0.005), computer science (*b* = 0.084, *t*(431) = 2.7, *p* = 0.008), biology (*b* = −0.072, *t*(431) = −2.5, *p* = 0.015), and engineering (*b* = −0.099, *t*(431) = 3.1, *p* = 0.002) with the STEM acronym suggesting that boys who associated computer science and engineering more with the STEM acronym had higher academic elective intentions for STEM and boys who associated mathematics and biology more with the STEM acronym had lower academic elective intentions for STEM.

**TABLE 1:** Association of subject areas with the STEM acronym and academic elective intentions for STEM.

	Association of subject areas with the STEM acronym					
	Mathematics	Physics	Computer science	Chemistry	Biology	Engineering
Girls and boys	−0.034 *(p = 0.28)*	−0.008 *(p = 0.81)*	0.043 *(p = 0.17)*	−0.025 *(p = 0.44)*	−0.022 *(p = 0.49)*	0.046 *(p = 0.14)*
Girls	0.035 *(p = 0.40)*	−0.056 *(p = 0.18)*	−0.018 *(p = 0.66)*	0.010 *(p = 0.81)*	0.046 *(p = 0.27)*	−0.032 *(p = 0.44)*
Boys	−0.136 (p = 0.005**)	0.062 *(p = 0.20)*	0.128 (p = 0.008**)	−0.074 *(p = 0.13)*	−0.117 (p = 0.015*)	0.149 (p = 0.002**)
Difference	0.171 (p = 0.007**)	−0.117 (*p = 0.065* ^a^ *)*	−0.146 (p = 0.021*)	0.084 *(p = 0.19)*	0.163 (p = 0.010*)	−0.180 (p = 0.004**)

*Note*: The table lists the correlation coefficients (*r*) between the association of subject areas with the STEM acronym (ranks of subject area) and the academic elective intentions for STEM. Correlation coefficients were tested against null (*ρ* = 0). The differences between girls (*n* = 582) and boys (*n* = 433) were compared on Fisher *z*‐transformed correlation coefficients. The *p* values are given in parenthesis (italicized).

^a^ *p* ≤ 0.1; *p ≤ 0.05; **p ≤ 0.01.

We also examined whether associations of STEM subject areas with the STEM acronym predicted students’ STEM‐profile choices at school by simple logistic regressions. When girls and boys were analyzed together, STEM‐profile choices were significantly predicted by associations of computer science (*χ* ^2^(1) = 14.0, *p* < 0.001, *R* ^2^ = 0.02, AUC = 0.565) and chemistry (*χ* ^2^(1) = 6.2, *p* = .013, *R* ^2^ = 0.01, AUC = 0.546) although the effects (as indicated by *R* ^2^ or AUC) were small. When only girls were analyzed, associations of subject areas with the STEM acronym did not predict STEM‐profile choices at school (all *p* ≥ 0.071). However, for boys, association of mathematics (*χ* ^2^(1) = 9.1, *p* = 0.003, *R* ^2^ = 0.03, AUC = 0.590), computer science (*χ* ^2^(1) = 4.9, *p* = 0.027, *R* ^2^ = 0.02, AUC = 0.573), and engineering (*χ* ^2^(1) = 9.0, *p* = 0.003, *R* ^2^ = 0.03, AUC = 0.583) predicted STEM‐profile choices although the effects (as indicated by *R* ^2^ or AUC) were small.

In summary, the association of subject areas with the STEM acronym did not significantly affect girls’ elective intentions for STEM or their STEM‐profile choices at school. However, boys who associated mathematics or biology more with STEM had lower academic elective intentions for STEM, and boys who associated computer science or engineering more with STEM had higher academic elective intentions for STEM and were more likely to choose a STEM profile at school.

### Value beliefs for STEM and their relation to academic elective intentions for STEM and STEM‐profile choices at school

We also investigated the relationship between value beliefs for STEM and academic elective intentions for STEM and STEM‐profile choices. As our scale analysis showed that the scale for value beliefs was comprised of two subscales (valence and cost), the relationship was examined for each (Table 2). When girls and boys were analyzed together, the valence subscale was significantly positively correlated with academic elective intentions for STEM, suggesting that the more students considered STEM intrinsically valuable, personally important, or useful, the higher their academic elective intentions for STEM were. The cost subscale was negatively correlated with academic elective intentions for STEM, suggesting that students who associated STEM with lower costs had higher academic elective intentions for STEM. This pattern was also confirmed by a multiple regression. Academic elective intention scores for STEM were significantly predicted by value beliefs (*adj‐R* ^2^ = 0.57, *F*(2, 1019) = 686, *p* < 0.001). Both subscales, valence (*b* = 0.781, *t* = 32.5, *p* < 0.001) and cost (*b* = −0.145, *t* = −6.7, *p* < 0.001), significantly contributed to the prediction.

**TABLE 2:** Value beliefs and academic elective intentions for STEM.

	Value beliefs	
	Valence	Cost
Girls and boys	0.745 (p < 0.001***)	−0.364 (p < 0.001***)
Girls	0.755 (p < 0.001***)	−0.466 (p < 0.001***)
Boys	0.735 (p < 0.001***)	−0.220 (p < 0.001***)
Difference	0.020 *(p = 0.49)*	−0.246 (p < 0.001***)

*Note*: The table lists the correlation coefficients (*r*) between the two subscales of value beliefs (valence and cost) and the scores in academic elective intentions for STEM. Correlation coefficients were tested against null (*ρ* = 0). The differences between girls (*n* = 585) and boys (*n* = 437) were compared on Fisher *z*‐transformed correlation coefficients. The *p* values are given in parenthesis (italicized).

^***^ *p* ≤ 0.001.

Similar relationships were found when girls and boys were analyzed separately. The valence subscale significantly and positively correlated with academic elective intentions for STEM in both girls and boys. Moreover, the cost subscale significantly and negatively correlated with academic elective intentions for STEM, although this correlation was significantly stronger for girls than boys (Table 2). Multiple regression analyses also confirmed this pattern. For girls, value beliefs significantly predicted academic elective intentions for STEM (*adj‐R* ^2^ = 0.59, *F*(2, 582) = 424, *p* < 0.001), and both the valence (*b* = 0.776, *t* = 23.2, *p* < 0.001) and the cost (*b* = −0.175, *t* = −5.8, *p* < 0.001) subscales contributed significantly to the prediction. For boys, academic elective intentions for STEM were also predicted by value beliefs (*adj‐R* ^2^ = 0.55, *F*(2, 434) = 271, *p* < 0.001), and both the valence (*b* = 0.783, *t* = 22.3, *p* < 0.001) and the cost (*b* = −0.125, *t* = −3.8, *p* < 0.001) subscales contributed significantly to the prediction.

Value beliefs also predicted students’ STEM‐profile choices at school. When girls and boys were analyzed together, a multiple logistic regression showed that students’ STEM‐profile choices were significantly predicted by value beliefs (*χ* ^2^(2) = 231.8, *p* < 0.001, *R* ^2^ = 0.27, AUC = 0.772) and both subscales, valence (*B = *0.93, *OR* = 2.53, *Z* = 10.9, *p* < 0.001) and cost (*B =* −0.39, *OR* = 0.68, *Z* = 5.5, *p* < 0.001), contributed to the prediction.

**FIGURE 2:**
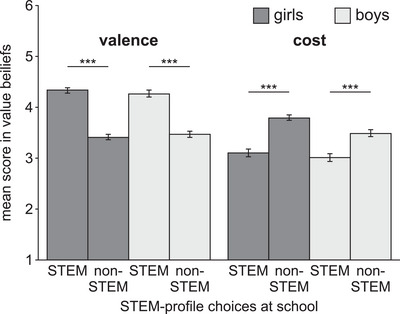
Value beliefs by STEM‐profile choices at school. Bars show the mean scores of the two subscales of value beliefs (valence and cost) separately for STEM‐profile choices (STEM profile vs. non‐STEM profile) and separately for girls (*n* = 643) and boys (*n* = 478). Differences between groups (STEM vs. non‐STEM) were compared by unpaired *t*‐tests. Error bars show the standard error of the mean. ***p ≤ 0.001.

Similar relationships were found when both groups were analyzed separately (Figure 2). Girls and boys who chose a STEM profile at school had higher mean scores in the valence subscale and lower mean scores in the cost subscale than students who chose a different profile. Correspondingly, multiple logistic regressions showed for girls that STEM‐profile choices were predicted by value beliefs (*χ* ^2^(2) = 132.3, *p* < 0.001, *R* ^2^ = 0.28, AUC = 0.779) and both subscales, valence (*B = 1*.00, *OR* = 2.72, *Z* = 7.9, *p* < 0.001) and cost (*B =* −0.31, *OR* = 0.74, *Z* = 3.0, *p* = 0.002) contributed to the prediction. For boys, STEM‐profile choices at school were also predicted by value beliefs (*χ* ^2^(2) = 88.6, *p* < 0.001, *R* ^2^ = 0.25, AUC = 0.758) and both subscales, valence (*B = *0.90, *OR* = 2.45, *Z* = 7.3, *p* < 0.001) and cost (*B =* −0.40, *OR* = 0.67, *Z* = 3.9, *p* < 0.001) contributed to the prediction.

In summary, value beliefs for STEM were strongly related to students’ academic elective intentions for STEM and STEM‐profile choices at school. For both girls and boys, high scores in the valence subscale and low scores in the cost subscale were associated with higher academic elective intentions for STEM and STEM‐profile choices at school.

### Associations of subject areas with the STEM acronym and their relation to value beliefs for STEM

Next, we examined the relationships between the associations of subject areas with the STEM acronym and the two subscales of value beliefs for STEM (Table 3). When girls and boys were analyzed together, there was one significant positive correlation between associations of mathematics with the STEM acronym and the cost subscale and one significant negative correlation between the association of engineering and the cost subscale. Simple regressions showed that cost scores were predicted by the association of STEM with mathematics (*b* = 0.052, *t*(1013) = 2.6, *p* = 0.010) and engineering (*b* = −0.043, *t*(1013) = −2.0, *p* = 0.048), suggesting that students who associated STEM most strongly with mathematics considered STEM more costly, and students who associated STEM most strongly with engineering considered STEM less costly. No other associations of school subjects with the STEM acronym showed a significant relationship (all *p* ≥ 0.37). No significant correlations or regression coefficients were observed for the valence subscale (all *p* ≥ 0.07).

**TABLE 3:** Association of subject areas with the STEM acronym and value beliefs.

	Association of subject areas with the STEM acronym					
	Mathematics	Physics	Computer science	Chemistry	Biology	Engineering
*Valence*						
Girls and boys	−0.001 *(p = 0.97)*	0.020 *(p = 0.52)*	0.046 *(p = 0.14)*	−0.056 *(p = 0.072^a^)*	−0.016 *(p = 0.61)*	0.005 *(p = 0.87)*
Girls	0.058 *(p = 0.16)*	−0.034 *(p = 0.41)*	−0.032 *(p = 0.44)*	−0.028 *(p = 0.50)*	0.082 (p = 0.047*)	−0.067 *(p = 0.11)*
Boys	−0.087 *(p = 0.071^a^)*	0.105 (p = 0.029*)	0.127 (p = 0.008**)	−0.084 *(p = 0.079^a^)*	−0.131 (p = 0.006**)	0.085 *(p = 0.079^a^)*
Difference	0.145 (p = 0.023*)	−0.139 (p = 0.028*)	−0.159 (p = 0.012*)	0.056 *(p = 0.38)*	0.213 (p < 0.001***)	−0.152 (p = 0.017*)
*Cost*						
Girls and boys	0.081 (p = 0.010**)	−0.012 *(p = 0.70)*	−0.028 *(p = 0.37)*	0.026 *(p = 0.41)*	−0.009 *(p = 0.78)*	−0.062 (p = 0.048*)
Girls	0.043 *(p = 0.30)*	0.049 *(p = 0.24)*	−0.022 *(p = 0.59)*	0.025 *(p = 0.55)*	−0.015 *(p = 0.72)*	−0.077 *(p = 0.065^a^)*
Boys	0.143 (p = 0.003**)	−0.119 (p = 0.013*)	0.015 *(p = 0.76)*	0.006 *(p = 0.91)*	−0.040 *(p = 0.41)*	−0.021 *(p = 0.67)*
Difference	−0.100 *(p = 0.11)*	0.169 (p = 0.008**)	−0.037 *(p = 0.562)*	0.019 *(p = 0.77)*	0.025 *(p = 0.69)*	−0.056 *(p = 0.38)*

*Note*: The table lists the correlation coefficients (*r*) between the association of subject areas with the STEM acronym ranks and the two subscales of value beliefs (valence and cost). Correlation coefficients were tested against null (*ρ* = 0). The differences between girls (*n* = 582) and boys (*n* = 433) were compared on Fisher *z*‐transformed correlation coefficients. The *p* values are given in parenthesis (italicized).

*^a^p* ≤ 0.1; *p ≤ 0.05; **p ≤ 0.01; ***p ≤ 0.001.

However, when both groups were analyzed separately, a more specific pattern emerged. For girls, a simple regression showed that associations of biology with the STEM acronym (*b* = 0.046*, t*(580) = 2.0, *p* = 0.047) significantly predicted scores in the valence subscale suggesting that girls who associated biology with the STEM acronym assessed STEM as more intrinsically valuable, personally meaningful, or useful. No significant correlation or regression coefficient was observed for the cost subscale (all *p* ≥ 0.07).

A different pattern emerged for boys. The valence subscale correlated negatively with associations of biology and positively with associations of physics or computer science. Simple regressions showed that associations of biology (*b* = −0.074, *t*(431) *=* −2.7, *p* = 0.011), physics (*b* = 0.073, *t*(431) = 2.2, *p* = 0.029), and computer science (*b* = 0.077, *t*(431) = 2.7, *p* = 0.008) with the STEM acronym significantly predicted scores in the valence subscale. This relationship suggests that the less boys associated biology with the STEM acronym and the more they associated physics or computer science with the STEM acronym, the more they assessed STEM as intrinsically valuable, personally meaningful, and useful. Simple regressions for predicting scores in the cost subscale showed that they were significantly predicted by associations of mathematics (*b* = 0.091, *t*(431) *=* 3.0, *p* = 0.003) and physics (*b* = −0.089, *t*(431) = −2.5, *p* = 0.013), suggesting that the more boys associated mathematics with the STEM acronym and the less with physics, the more costly they assessed STEM. No other significant correlation or regression coefficient was observed for the cost subscale (all *p* ≥ 0.41).

In summary, associations of subject areas with the STEM acronym were related to value beliefs in STEM. Across girls and boys, a strong association of mathematics with the STEM acronym was related to assessing STEM as more costly. However, girls and boys also differed. A strong association of biology with the STEM acronym was positively related to the valence subscale for girls but negatively for boys. For boys only, associations of physics with the STEM acronym were positively related to valence and negatively related to cost, and associations of computer science were positively related to valence.

### Associations of subject areas with the STEM acronym and their relation to elective intentions for STEM and STEM‐profile choices, mediated by value beliefs

So far, our analyses showed that associations of subject areas with the STEM acronym were related to value beliefs for STEM, and value beliefs for STEM were related to academic elective intentions for STEM and STEM‐profile choices. Hence, it is possible that associations of subject areas with the STEM acronym indirectly affect academic elective intentions for STEM and STEM‐profile choices at school via value beliefs rather than directly. This assumption was tested by path analyses with a mediator model.

**FIGURE 3:**
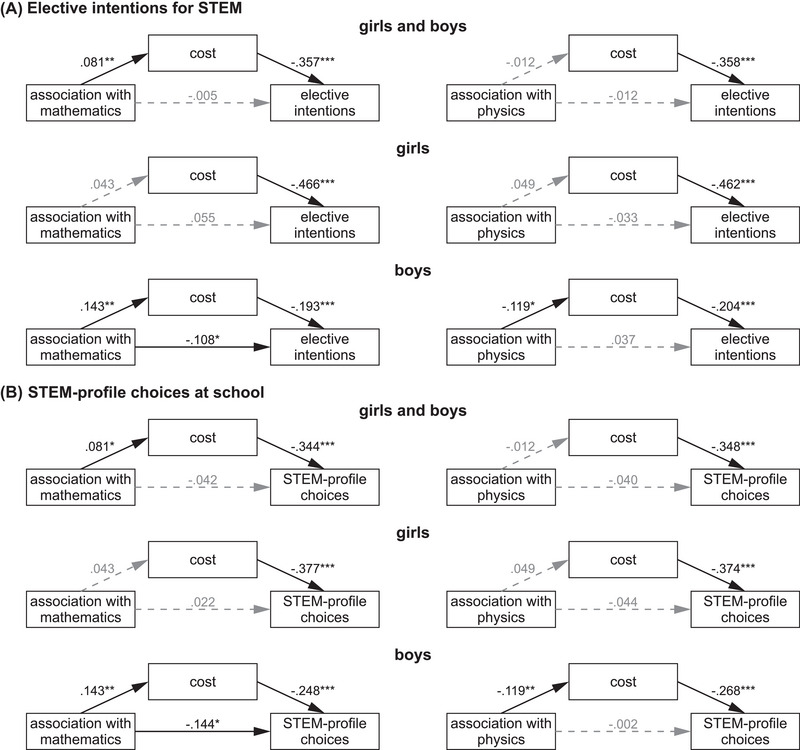
Path models for predicting academic elective intentions for STEM and STEM‐profile choices at school, mediated by the cost subscale of value beliefs. The mediation models tested whether students’ association of subject areas with the STEM acronym predict (A) academic elective intentions for STEM or (B) STEM‐profile choices at school, either directly or mediated (indirectly) via the subscale cost of value beliefs. The analyses were performed for both groups (girls and boys) together (top row) and separately for girls (middle) and boys (bottom). Path coefficients (*b*) were tested by Sobel Z tests. Nonsignificant paths are indicated by dashed gray lines and gray coefficients. Only models with at least one significant indirect path for at least one group or outcome measure are shown. See also Tables S5 and S6. *p ≤ 0.05; **p ≤ 0.01; ***p ≤ 0.001.

First, we tested whether the cost subscale of value beliefs served as a mediator between students’ associations of subject areas with the STEM acronym and the outcome variables (i.e., academic elective intentions for STEM and STEM‐profile choices). Because of the collinearity across the association rankings, the association of each subject area with the STEM acronym was tested separately. When girls and boys were analyzed together, there was a significantly negative indirect (mediated) effect of associations of mathematics on academic elective intentions for STEM mediated by cost (Figure 3A) (*b =* −0.016, *Sobel Z* = −2.5, *p* = 0.011). This indirect effect reflects a positive relationship (*b = *0.081) between associations of mathematics with the STEM acronym and the cost subscale, which in turn was negatively related (*b =* −0.357) to academic elective intentions for STEM. No other school subjects showed a significant mediation effect, but there was a marginally significant (*p* = 0.051) positive indirect effect for engineering (Table S5). No significant direct effects were observed.

The association of mathematics with the STEM acronym also showed a significant negative indirect effect (via the mediator cost) in predicting STEM‐profile choices (Figure 3B) (*b =* −0.028, *Z* = −2.4, *p* = 0.014). Again, strong associations of mathematics with the STEM acronym were associated with higher scores in the cost subscale (*b = *0.081), which in turn were associated with a lower probability for STEM‐profile choices (*b =* −0.344). No other school subjects showed a significant mediation effect, but there was a marginally significant (*p* = 0.055) positive indirect effect for engineering (Table S6). In addition, two direct effects between associations and STEM‐profile choices were observed. There was a negative relationship between associations of chemistry (*b =* −0.090, *Z* = −2.3, *p* = 0.020) and a positive relationship between associations of engineering (*b =* 0.127, *Z* = 3.3, *p* < 0.001) with STEM‐profile choices.

When girls and boys were analyzed separately, no significant indirect effects via the mediator cost were observed in girls for any outcome variables (academic elective intentions for STEM and STEM‐profile choices). However, there were a marginally significant negative direct (*p* = 0.065) and a positive indirect (*p* = 0.067) effect for associations of engineering and elective intentions for STEM and a marginally significant positive indirect (*p* = 0.067) effect between associations of engineering and STEM‐profile choices (Tables S5 and S6). For boys, there were significant indirect effects of associations with the STEM acronym for mathematics (*b =* −0.016, *Z* = −2.4, *p* = 0.015) and physics (*b =* 0.017, *Z* = 2.2, *p* = 0.031) on academic elective intentions mediated by cost (Figure 3A), indicating that STEM was perceived as more costly if boys associated mathematics (*b =* 0.143) and less costly if they associated physics (*b =* −.119) with the STEM acronym; and cost scores were negatively related to elective intentions for STEM. Similarly, STEM‐profile choices were indirectly (mediated by cost) affected by associations with the STEM acronym for mathematics (*b =* −0.036, *Z* = −2.5, *p* = 0.012) and physics (*b =* 0.032, *Z* = 2.3, *p* = 0.020) (Figure 3B). Moreover, several direct effects between associations with the STEM acronym of mathematics (*b =* −0.062, *Z* = −2.3, *p* = 0.022), computer science (*b =* 0.077, *Z* = 2.8, *p* = 0.005), biology (*b =* −0.070, *Z* = −2.7, *p* = 0.007), and engineering (*b =* 0.087, *Z* = 3.1, *p* = 0.002) and elective intentions for STEM (Table S5) were observed. Similarly, direct effects were also observed between associations of mathematics (*b =* −0.147, *Z* = −2.5, *p* = 0.013), computer science (*b =* 0.139, *Z* = 2.3, *p* = 0.020), and engineering (*b =* 0.176, *Z* = 3.0, *p* = 0.003) and STEM‐profile choices (Table S6). These direct effects suggest that the more boys associated computer science or engineering and the less they associated mathematics or biology with the STEM acronym, the higher their academic elective intentions for STEM were, and the more likely they were to choose a STEM profile.

Next, we tested whether the valence subscale of value beliefs was a mediator between students’ associations of subject areas with the STEM acronym and the outcome variables (academic elective intentions for STEM and STEM‐profile choices at school). When girls and boys were analyzed together, there was no significant indirect effect between associations of subject areas with the STEM acronym and the two outcome variables (Tables S5 and S6). There was only a marginally significant indirect effect between the association of chemistry with the STEM acronym and academic elective intentions for STEM (*p* = 0.072) and STEM‐profile choices (*p* = 0.071). Additionally, there were significant positive direct effects between the association of engineering and academic elective intentions (*b =* 0.026, *Z* = 2.0, *p* = 0.045) or STEM‐profile choices (*b =* 0.145, *Z* = 4.0, *p <* 0.001). There were also two marginally significant negative direct effects between associations of mathematics (*p* = 0.055) and chemistry (*p* = 0.058) on STEM‐profile choices. No other indirect or direct effects were significant.

**FIGURE 4:**
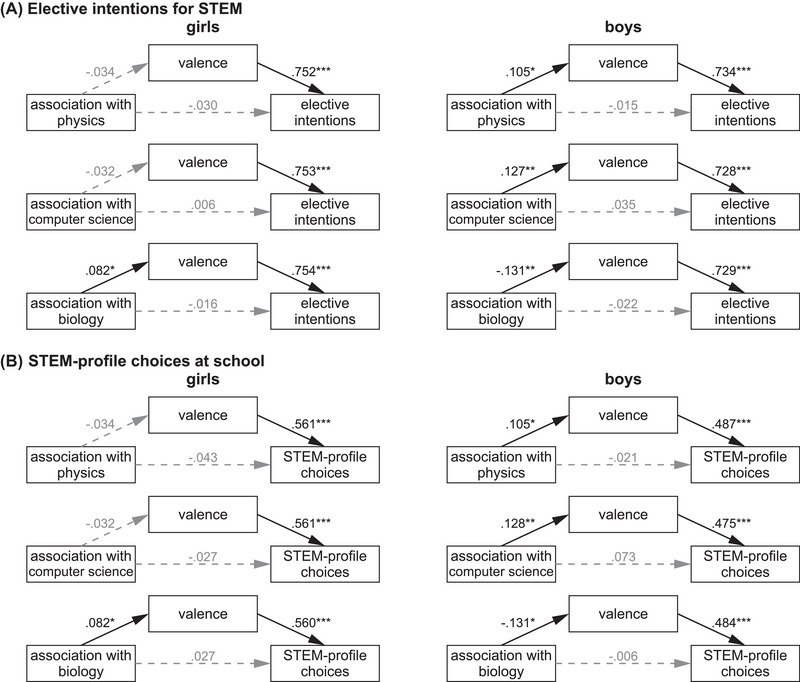
Path models for predicting academic elective intentions for STEM and STEM‐profile choices at school by associations of subject areas with the acronym STEM, mediated by the subscale valence of value beliefs. The mediation models tested whether the subject area association ranks with the acronym STEM predicted (A) academic elective intentions for STEM or (B) STEM‐profile choices at school, either directly or mediated (indirectly) via the subscale valence of value beliefs. Data for girls (left) and boys (right) were analyzed separately as the mediation effects differed between groups (see text). Path coefficients (*b*) were tested by Sobel Z tests. Nonsignificant paths are indicated by dashed gray lines and gray coefficients. Only models with at least one significant indirect path for at least one group or outcome measure are shown. See also Tables S5 and S6. *p ≤ 0.05; **p ≤ 0.01; ***p ≤ 0.001.

However, when girls and boys were examined separately, there were distinct mediation effects: For girls, there was a significant positive indirect effect of associations of biology with the STEM acronym on academic elective intentions for STEM, mediated by valence (*b =* 0.034, *Z* = 2.0, *p* = 0.047) (Figure 4A). This indirect effect reflects a positive relationship (*b = *0.082) between biology associations with the STEM acronym and valence, which positively affected academic elective intentions for STEM (*b* = 0.754). A similar positive mediation effect was observed for associations of biology on STEM‐profile choices (*b =* 0.046, *Z* = 2.0, *p* = 0.050) (Figure 4B). The more girls associated biology with STEM, the higher their valence ratings (*b = *0.082), and the higher their valence ratings, the more likely they chose a STEM profile (*b* = 0.560). Moreover, one positive direct effect between the association of engineering with the STEM acronym and STEM‐profile choices (*b =* 0.134, *Z* = 2.9, *p* = 0.004) was observed (Table S6), suggesting that the more girls associated engineering with STEM, the more likely they chose a STEM profile. No other direct or indirect effects were significant.

For boys, the mediation effects differed from those in girls. We found a significantly negative indirect effect of biology associations with the STEM acronym on academic elective intentions, mediated by valence (*b =* −0.053, *Z* = −2.7, *p* = 0.006) (Figure 4A). This indirect effect reflects a negative relationship (*b =* −0.131) between biology associations with the STEM acronym and valence, which affected academic elective intentions for STEM (*b = *0.729). A similar mediation effect was observed between associations of biology and STEM‐profile choices at school (*b =* −0.064, *Z* = −2.4, *p* = 0.016) (Figure 4B). Associations of biology with the STEM acronym negatively affected valence ratings (*b =* −0.131), which in turn were positively related to STEM‐profile choices (*b = *0.484). This suggests that the more boys associated the STEM acronym with biology, the lower their valence of STEM ratings, which was associated with lower elective intentions for STEM and fewer choices of a STEM profile. On the other hand, there were positive indirect effects between associations of physics (*b =* 0.052, *Z* = 2.2, *p* = 0.029) or computer science (*b =* 0.055, *Z* = 2.7, *p* = 0.008) with the STEM acronym and academic elective intentions, mediated by valence (Figure 4A). These indirect effects reflect a positive relationship between associations of physics (*b = *0.105) or computer science (*b = *0.127) and the valence ratings, which positively affected academic elective intentions for STEM. This suggests that the more boys associated the STEM acronym with physics or computer science, the higher they assessed the valence of STEM, which was associated with higher academic elective intentions for STEM. Very similar mediation effects were observed between associations of physics (*b =* 0.051, *Z* = 2.2, *p* = 0.030) or computer science (*b =* 0.061, *Z* = 2.6, *p* = 0.011) and STEM‐profile choices at school (Figure 4B). Associations of physics (*b = *0.105) or computer science (*b = *0.128) with the STEM acronym positively affected valence ratings, which in turn were positively related to STEM‐profile choices. Indirect effects for associations of other school subjects, such as mathematics, chemistry, or engineering, were only marginally significant (Tables S5 and S6). However, associations of mathematics with STEM showed a significant negative direct effect on academic elective intentions for STEM (*b =* −0.042, *Z* = −2.2, *p* = 0.026) and STEM‐profile choices (*b =* −0.141, *Z* = −2.4, *p* = 0.015), and associations of engineering with STEM showed a positive direct effect on academic elective intentions for STEM (*b =* 0.052, *Z* = 2.7, *p* = 0.007) and STEM‐profile choices (*b =* 0.141, *Z* = 2.5, *p* = 0.013). No other direct effects were significant.

In summary, the association of subject areas with the STEM acronym affected students’ academic elective intentions for STEM and STEM‐profile choices at school primarily indirectly via value beliefs (i.e., the intrinsic value, personal importance, and utility ascribed to STEM) and cost beliefs (i.e., the costs or effort ascribed to STEM) rather than directly. Mediation effects for cost beliefs were observed for associations of mathematics (girls and boys together) and physics (only boys). The more students associated mathematics with the STEM acronym (and the less with physics), the more costly they assessed STEM, and the lower their academic elective intentions for STEM and the probability of choosing a STEM profile at school. Mediation effects involving the valence subscale as a mediator were substantially different for girls and boys. For girls, high biology associations with the STEM acronym were associated with higher value beliefs for STEM and, consequently, higher academic elective intentions for STEM and STEM‐profile choices at school. For boys, low associations of biology with the STEM acronym and high associations of physics or computer science with the STEM acronym were associated with higher valence for STEM and, consequently, higher academic elective intentions for STEM and STEM‐profile choices at school.

## DISCUSSION

Campaigns to encourage more skilled people and to inspire girls and women for STEM fields often adopt the STEM acronym to promote courses, university programs, and careers. However, there is little research on whether, and under what conditions, such broad‐brushed campaigns can be effective. A minimum requirement is that the addressed group is familiar with the STEM acronym. However, even if the target audience is familiar with the STEM acronym, it can be assumed that campaigns will have different effects depending on which STEM subject areas the target groups associate with the STEM acronym. Initial evidence suggests that high school students are more interested in STEM if they assume that the STEM acronym also includes medicine.^30^ However, no study systematically investigates which STEM subject areas the STEM acronym is most strongly associated with, what relevance these associations have for STEM choices, and their predictors. In our study, we addressed this research gap by investigating six research questions related to individuals’ awareness of and associations with the German equivalent of the STEM acronym (MINT).

Our first research question examined whether eighth graders know the STEM acronym. Interestingly, more than a quarter of the students (28%) did not know the STEM acronym. This is concerning, given that the STEM acronym has been used for decades to advertise STEM university degrees, STEM professions, and extracurricular STEM programs. An essential first step in attracting students to such programs is, thus, to ensure that they understand what STEM offerings refer to. Our results are particularly concerning, considering that the STEM acronym was presented in writing in our study, and all attention was focused on the acronym. As STEM programs are often advertised via the spoken word, for example, on podcasts, on the radio, or by community members, it must be assumed that such approaches reach even fewer people because comprehension of words can differ when reading and when listening.^48^, ^49^ For example, even if the STEM acronym is known, fewer people will listen attentively when offerings are being discussed verbally since people may engage in other tasks while listening, which leads to decreased comprehension compared to focused reading.^50^ Future research should investigate the advantages and disadvantages of different presentation modalities of the STEM acronym or STEM offerings.

Interestingly, in our study, girls (79%) were more familiar with the STEM acronym than boys (63%), despite boys having a significantly higher affinity for STEM.^26^ This finding seems to contradict notions that girls in Germany avoid STEM because they are unfamiliar with the concept. Instead, the finding supports ideas described in earlier studies: Girls are familiar with STEM domains but categorize them as typically male^51^ and, therefore, feel a low sense of belonging.^52^

Our second research question examined which STEM subject areas students most strongly associate with the STEM acronym. Our survey included mathematics, all science subjects taught at school (biology, chemistry, physics), computer science, and engineering. The students associated the STEM acronym most strongly with mathematics. The strongest association of the STEM acronym with mathematics could be because the German equivalent, MINT, begins with the letter “M,” which stands for mathematics. This might be different in English, for example, where the acronym starts with an “S,” which stands for science. Another reason might be that eighth‐grade students have already had math classes since elementary school, while other science subjects (e.g., biology, chemistry, and physics) are typically introduced in later grades. Computer science and engineering are not mandatory school subjects in Germany, and only some schools offer them. Future research could investigate the order of the letters in the STEM acronym (across countries) and prior exposure of the subjects in the school curricula.

We also found gender differences in the associations of the STEM acronym with the different STEM subject areas. Girls associated the STEM acronym more strongly with natural science subjects (physics, chemistry, and biology) than boys. By contrast, boys associated the STEM acronym more strongly with the subject areas known from a professional context where women are particularly underrepresented in Germany, namely, computer science and engineering.^53^ Future research should examine whether similar patterns can be found in other countries.

The remaining four research questions addressed how the associations of the STEM acronym with different subject areas affect students’ career decisions. In Research Question 3, we examined whether students’ associations of STEM subject areas with the STEM acronym are related to academic elective intentions for STEM and STEM‐profile choices. No significant relationships were found for girls. However, boys who associated mathematics or biology more strongly with the STEM acronym reported lower academic elective intentions for STEM. In contrast, boys who associated computer science or engineering more strongly with the STEM acronym reported higher academic elective intentions for STEM and were more likely to choose STEM in school. These results have implications for STEM promotion campaigns, particularly regarding which subjects should be emphasized and which should not. At the same time, the findings indicate which subjects’ reputations need to be improved in order to become more attractive to boys.

Research Question 4 examined the relationships between value beliefs and academic elective intentions for STEM and STEM‐profile choices at school. Consistent with other studies,^1^, ^34^, ^35^ we found strong relationships between these variables. For girls and boys, high valence and low‐cost assessments of STEM were associated with higher academic elective intentions for STEM and STEM‐profile choices at school. The opposing effects of valence and cost beliefs suggest that it seems insufficient for advertising campaigns and interventions to focus solely on increasing the intrinsic value, attainment value, and utility value of STEM. Our findings show that STEM activities and tasks have both benefits and costs. So far, only a few studies have distinguished between these two main components of value beliefs.^24^, ^26^ Future research should consider and distinguish various facets of the valence component (intrinsic value, attainment value, and utility value) and the cost component (effort cost, opportunity cost, and emotional cost).^27^ In particular, qualitative studies could provide insights into which valence and cost facets are essential for STEM choices.

Research Question 5 addressed the relationships between associations of subject areas with the STEM acronym and value beliefs in STEM. Again, we considered the valence and cost components of value beliefs separately and found that these relationships differed across subject areas and between girls and boys. For both girls and boys, a strong association of mathematics with the STEM acronym was related to assessing STEM as more costly. Girls who associated the STEM acronym strongly with biology reported a higher valence of STEM, whereas for boys, a strong association of the STEM acronym with biology related to negative valence assessments. Furthermore, a strong association of the STEM acronym with physics and computer science related positively to valence assessments and negatively to cost assessments of STEM for boys but not girls.

Our findings are consistent with other studies examining value beliefs for different subject areas.^26^, ^54^ However, our study is the first to comprehensively cover different STEM subject areas and examine relations between associations of these STEM subject areas with the STEM acronym and value beliefs. Interestingly, the strength of the association of the STEM acronym with different STEM subject areas is related to general STEM value beliefs. In other words, depending on the subject area with which students most strongly associate the STEM acronym, the reputation of STEM (operationalized via value beliefs) seems to be perceived as more or less positive. Similar to other studies,^26^, ^54^ biology seems to have a particularly good reputation among girls, while physics and computer science have a particularly good reputation among boys. These results suggest that promotional efforts broadly targeting STEM affect boys and girls differently. It might be essential to develop targeted campaigns for each group separately (e.g., focusing on different subject areas). Mathematics requires special attention as it is associated with high costs for both genders.

In Research Question 6, we investigated whether associations of subject areas with the STEM acronym and their relations to elective intentions for STEM and STEM‐profile choices are mediated by value beliefs. In summary, the mediation analyses suggest that the subject areas that students associate with the STEM acronym affect academic elective intentions for STEM and STEM choices at school primarily indirectly via value beliefs (i.e., the intrinsic value, personal importance, and utility ascribed to STEM) and cost beliefs (i.e., the costs or effort ascribed to STEM) rather than directly. Mediation effects for cost beliefs were observed for associations of mathematics (girls and boys combined) and physics (only boys). The more students associated mathematics with the STEM acronym (and the less boys associated it with physics), the more costly they assessed STEM, and the lower their academic elective intentions for STEM and the probability of choosing a STEM profile in school. We also found mediation effects for valence for associations of biology, physics, and computer science with the STEM acronym. These were substantially different for girls and boys. For girls, high biology associations with the STEM acronym were related to higher value beliefs for STEM and consequently with higher academic elective intentions for STEM and STEM‐profile choices in school. For boys, low associations of biology with the STEM acronym and high associations of physics or computer science with the STEM acronym were associated with higher value beliefs for STEM and, consequently, higher academic elective intentions for STEM and STEM‐profile choices in school.

Although our results do not explain gender differences in STEM, they reveal which STEM subject areas boys and girls associated with the STEM acronym and how these associations influence their elective intentions for STEM and STEM choices directly and indirectly through value beliefs. This finding substantially exceeds previous studies on value beliefs, which analyzed individual STEM subject areas.^24^, ^26^ Our study highlights the importance of investigating associations of the STEM acronym with individual STEM subject areas. Our results raise several further research questions. Future research should further distinguish various facets of the cost component. Furthermore, extensive investigations of the other variables included in the situated expectancy–value theory^27^ would be desirable, such as the second proximal variable (outcome expectancy) that predicts STEM choices in addition to values. Likewise, other distal variables of the expectancy–value theory need to be assessed to fully understand the mechanisms behind associations between subject areas with the STEM acronym and STEM choices.

Our study has several limitations. As indicated in the Methods section, students’ associations with the STEM acronym were assessed by a forced‐ranking procedure (rather than a rating procedure). Although rankings are only feasible with small item sets, they have several advantages compared to ratings. Rankings reduce response biases (e.g., straightlining, center tendency, leniency, stringency), rankings avoid ties, and rankings tend to have higher discriminating power and reliability.^55^ However, rankings also come with special assessment characteristics. Rankings provide ipsative scores (unlike ratings, which provide normative scores). As a consequence, the rank measurements for the various subject areas were partially statistically dependent (multicollinear). However, we believe this limitation had a negligible effect on our main findings. Collinearity (if too strong) would have resulted in findings that each subject area correlated equally with value beliefs. Our main finding, however, shows distinct differences across subject areas. These differences cannot be attributed to collinearity. Finally, rankings (compared to ratings) minimize self‐reporting subjectivity as responses are referenced to the provided objective options (rather than to subjective internal standards).^56^ Although this might be a desirable characteristic as it reduces interindividual variation (while preserving intraindividual variation), it also has possibly undesirable consequences if the self‐reporting subjectivity contains meaningful information. Interindividual differences and group differences (girls vs. boys) might have been diminished because the mean rank across all subject areas was identical for all girls and boys. Hence, it is possible that the observed effects would be stronger if rating (rather than ranking) procedures were adopted.

We examined a German sample of eighth‐graders. It is possible that our results cannot be transferred to other age groups and countries. On the one hand, associations of the STEM acronym with different STEM subject areas could be influenced by the composition and the order of the letters in the acronym. The German analog of the STEM acronym, MINT, names domains that are partially different than those in the English STEM acronym. For example, it includes “Informatik” (computer science), which is not included in the STEM acronym. Moreover, it begins with an “M” for “Mathematik” (mathematics), which might have prompted associations with mathematics by a primacy effect. On the other hand, country‐specific school curricula could influence the associations. For example, it should make a difference whether STEM is an integrated school subject or whether individual STEM subject areas are taught separately. Furthermore, the acronym might be more strongly associated with subjects already taught at lower grades and thus have been taught for longer. Finally, it could also be influenced by how well‐known the STEM acronym is and whether countries strongly focus on individual STEM subject areas (e.g., those with a vast gender gap) in STEM promotion campaigns. Although it is too early to rule out these limitations, our findings nevertheless suggest that students’ perception of the STEM acronym is also relevant in other countries.

Furthermore, our sample shows several biases. Although the participation rate was acceptable and the ratio of participating girls to boys did not differ significantly from the ratio in participating schools, there was a school selection bias. Our sample was drawn from schools in primarily urban areas with a focus on STEM activities and from students of educated families. However, we believe that this selection bias does not compromise our main findings, as we would expect that those schools that focus on STEM education show fewer differences across school subjects. Hence, we would expect that other schools with less STEM interest would show even stronger differences than those observed in our sample.

Finally, while our findings suggest that value beliefs mediate the relationship between associations of the STEM acronym and elective intentions or STEM‐profile choices, it is important to acknowledge that these are not the only plausible mediators. In particular, self‐efficacy and identity may also drive STEM choices and activities.^57^ Self‐efficacy or identity is often conceptually and empirically intertwined with value beliefs.^28^ Including additional motivational factors would likely lead to a redistribution of explained variance across constructs rather than a linear increase.^58^ Our choice of mediators was theory‐driven, reflecting a focus on the value components of expectancy–value theory. Future studies could test broader models to examine how multiple motivational beliefs jointly contribute to STEM development.

## CONCLUSION

Our results provide the first clear evidence that the STEM acronym is perceived differently. Associations of the STEM acronym with different STEM subject areas are directly related to STEM elective intentions and STEM choices for boys and mediated by value beliefs for both boys and girls. Our results provide insights into the optimal design of advertising campaigns to attract people to STEM subjects. These findings suggest that promotional campaigns for broad audiences that use the STEM acronym and ignore these relationships likely lack effectiveness. On the one hand, not everyone is familiar with the acronym. On the other hand, such campaigns have very different effects depending on which STEM subjects are most strongly associated with the acronym. This indicates that advertising campaigns should focus on specific STEM subject areas instead of taking a broad approach to STEM.

## AUTHOR CONTRIBUTIONS

H.S. and A.Z. conceived the study and developed the theoretical framework. A.L.B. analyzed the data. H.S. took the lead in writing the manuscript. All authors provided critical feedback and approved the final manuscript.

## COMPETING INTERESTS

The authors declare no potential conflicts of interest with respect to the research, authorship, and/or publication of this article.

## Supporting information



Table S1 Demographics of the sample.
Table S2 Scale evaluation of value beliefs for STEM.
Table S3 Scale evaluation of academic elective intentions for STEM.
Table S4 Association of subject areas with the STEM acronym.
Table S5 Path models for predicting academic elective intentions for STEM by association of subject areas with the STEM acronym.
Table S6 Path models for predicting STEM‐profile choices at school by the association of subject areas with the STEM acronym.

## Data Availability

The datasets used and/or analyzed for this study are available from the corresponding author upon reasonable request.

## References

[1] Jansen, M. , Becker, M. , & Neumann, M. (2021). Dimensional comparison effects on (gendered) educational choices. Journal of Educational Psychology, 113(2), 330–350. 10.1037/edu0000524

[2] National Center for Education Statistics . (2024). Science, technology, engineering, and mathematics (STEM) education, by gender . https://nces.ed.gov/fastfacts/display.asp?id=899

[3] China Statistics Press . (2022). China Statistical Yearbook 2022 . https://www.stats.gov.cn/sj/ndsj/2022/indexeh.htm

[4] Federal State Statistics Service . (2022). *Russian Statistics* Yearbook 2022. https://eng.rosstat.gov.ru/storage/mediabank/Yearbook_2022.pdf

[5] OECD . (2024). New tertiary graduates in science, technology, engineering and mathematics as a share of new graduates. Organisation for Economic Co‐operation and Development. https://goingdigital.oecd.org/en/indicator/43

[6] Anger, C. , Betz, J. , Geis‐Thöne, W. , & Plünnecke, A. (2023). MINT‐Herbstreport 2023: Mehr MINT‐Lehrkräfte gewinnen, Herausforderungen der Zukunft meistern . https://www.iwkoeln.de/studien/christina‐anger‐julia‐betz‐wido‐geis‐thoene‐axel‐pluennecke‐mehr‐mint‐lehrkraefte‐gewinnen‐herausforderungen‐der‐zukunft‐meistern.html

[7] Xue, Y. , & Larson, R. C. (2015). Stem crisis or STEM surplus? Yes and yes. Monthly Labor Review, 10.21916/mlr.2015.14 PMC5800410 29422698

[8] Zaza, S. , Abston, K. , Arik, M. , Geho, P. , & Sanchez, V. (2020). What CEOs have to say: Insights on the STEM workforce. American Business Review, 23(1), 136–155. 10.37625/abr.23.1.136-155

[9] Mwenda, A. B. , Sullivan, M. , & Grand, A. (2019). How do Australian universities market STEM courses in YouTube videos? Journal of Marketing for Higher Education, 29(2), 191–208. 10.1080/08841241.2019.1633004

[10] Andrée, M. , & Hansson, L. (2013). Marketing the ‘Broad Line’: Invitations to STEM education in a Swedish recruitment campaign. International Journal of Science Education, 35(1), 147–166. 10.1080/09500693.2012.695880

[11] Kayan‐Fadlelmula, F. , Sellami, A. , Abdelkader, N. , & Umer, S. (2022). A systematic review of STEM education research in the GCC countries: Trends, gaps and barriers. International Journal of STEM Education, 9(1), 2. 10.1186/s40594-021-00319-7

[12] NASA . (2024). About NASA STEM engagement . https://www.nasa.gov/learning‐resources/stem‐engagement/

[13] Boy Scouts of America . (2024). STEM Nova Awards . https://www.scouting.org/stem‐nova‐awards/

[14] STEM Connector . We connect the world to the STEM workforce . Retrieved from https://www.stemconnector.com

[15] STEM Education Coalition . The largest, most unified coalition advocating for STEM . Retrieved from https://www.stemedcoalition.org/about

[16] STEM Next Opportunity Fund . (2022). Annual 2021 report . https://stemnext.org/wp‐content/uploads/2022/11/STEMNext_FY2021_Annual_Report.pdf

[17] DiscoverE . (2024). Transform a student's future . https://discovere.org

[18] FIRST . (2024). About FIRST . https://www.firstinspires.org/about

[19] Million Women Mentors . Million women mentors . Retrieved from https://www.millionwomenmentors.com

[20] National Girls Collaborative Project . (2024). About NGCP . https://ngcproject.org/about‐ngcp

[21] Varas, J. (2016). The native‐born STEM shortage . https://www.americanactionforum.org/wp‐content/uploads/2016/04/The‐Native‐Born‐STEM‐Shortage.pdf

[22] Yokoyama, H. M. , Ikkatai, Y. , McKay, E. , Inoue, A. , Minamizaki, A. , & Kano, K. (2024). Can affirmative action overcome STEM gender inequality in Japan? Expectations and concerns. Asia Pacific Business Review, 30(3), 543–559. 10.1080/13602381.2024.2320547

[23] Wang, M. ‑T. , & Degol, J. L. (2017). Gender gap in science, technology, engineering, and mathematics (STEM): Current knowledge, implications for practice, policy, and future directions. Educational Psychology Review, 29(1), 119–140. 10.1007/s10648-015-9355-x 28458499 PMC5404748

[24] Vinni‐Laakso, J. , Upadyaya, K. , & Salmela‐Aro, K. (2022). Associations between adolescent students' multiple domain task value‐cost profiles and STEM aspirations. Frontiers in Psychology, 13, 951309. 10.3389/fpsyg.2022.951309 36619107 PMC9815538

[25] Gaspard, H. , Dicke, A. ‑L. , Flunger, B. , Schreier, B. , Häfner, I. , Trautwein, U. , & Nagengast, B. (2015). More value through greater differentiation: Gender differences in value beliefs about math. Journal of Educational Psychology, 107(3), 663–677. 10.1037/edu0000003

[26] Gaspard, H. , Häfner, I. , Parrisius, C. , Trautwein, U. , & Nagengast, B. (2017). Assessing task values in five subjects during secondary school: Measurement structure and mean level differences across grade level, gender, and academic subject. Contemporary Educational Psychology, 48, 67–84. 10.1016/j.cedpsych.2016.09.003

[27] Eccles, J. S. , & Wigfield, A. A. (2020). From expectancy‐value theory to situated expectancy‐value theory: A developmental, social cognitive, and sociocultural perspective on motivation. Contemporary Educational Psychology, 61, 101859. 10.1016/j.cedpsych.2020.101859

[28] Wigfield, A. A. , & Eccles, J. S. J. S. (2000). Expectancy–value theory of achievement motivation. Contemporary Educational Psychology, 25(1), 68–81. 10.1006/ceps.1999.1015 10620382

[29] Breiner, J. M. , Harkness, S. S. , Johnson, C. C. , & Koehler, C. M. (2012). What is STEM? A discussion about conceptions of STEM in education and partnerships. School Science and Mathematics, 112(1), 3–11. 10.1111/j.1949-8594.2011.00109.x

[30] Adler, R. M. , Xu, M. , & Rittle‐Johnson, B. (2024). What counts as STEM, and does it matter? British Journal of Educational Psychology, 94(1), 165–180. 10.1111/bjep.12639 37907362

[31] Angier, N. (2010). STEM education has little to do with flowers. *The New York Times*. www.nytimes.com/2010/10/05/science/05angier.html

[32] Gaisch, M. , Rammer, V. , Sterrer, S. , & Takacs, C. (2023). Wie MINT gewinnt. Assoziationen, Erfolgsfaktoren und Hemmnisse österreichischer Schülerinnen in Bezug auf eine Ausbildung in den MINT‐Bereichen. Auftragsstudie für die MINTality Stiftung . https://pure.fh‐ooe.at/files/53451294/MINTality_Studie_Bericht_MINTgewinnt_FINAL.pdf

[33] Eccles (Parsons), J. S. , Adler, T. F. , Futterman, R. , Goff, S. B. , & Kaczala, C. M. (1983). Expectancies, values, and academic behaviors. In J. T. Spence (Ed.), Achievement and achievement motives (pp. 75–146). Freeman.

[34] Rosenzweig, E. Q. E. Q. , Wigfield, A. A. , & Eccles, J. S. (2022). Beyond utility value interventions: The why, when, and how for next steps in expectancy‐value intervention research. Educational Psychologist, 57(1), 11–30. 10.1080/00461520.2021.1984242

[35] Jiang, S. , Simpkins, S. D. , & Eccles, J. S. (2020). Individuals' math and science motivation and their subsequent STEM choices and achievement in high school and college: A longitudinal study of gender and college generation status differences. Developmental Psychology, 56(11), 2137–2151. 10.1037/dev0001110 32915052

[36] Wigfield, A. [A. ] , Rosenzweig, E. Q. [E. Q. ] , & Eccles, J. S. [J. S. ] (2017). Achievement values. In A. Elliot , & C. S. Dweck (Eds.), Handbook of competence and motivation (2nd ed., pp. 116–134). Guilford.

[37] Watt, H. M. G. , Bucich, M. , & Dacosta, L. (2019). Adolescents’ motivational profiles in mathematics and science: Associations with achievement striving, career aspirations and psychological wellbeing. Frontiers in Psychology, 10, 990. 10.3389/fpsyg.2019.00990 31316409 PMC6610331

[38] Lazarides, R. , Dicke, A. ‑L. , Rubach, C. , Oppermann, E. , & Eccles, J. S. (2021). Motivational profiles across domains and academic choices within Eccles et al.’s situated expectancy‐value theoretical framework. Developmental Psychology, 57(11), 1893–1909. 10.1037/dev0001250 34914452

[39] Umarji, O. , Wan, S. , Wolff, F. , & Eccles, J. (2023). The system of academic task values: The development of cross‐domain comparisons of values and college major choice. Developmental Psychology, 59(6), 1032–1044. 10.1037/dev0001519 37053390

[40] Olive, K. , Tang, X. , Loukomies, A. , Juuti, K. , & Salmela‐Aro, K. (2022). Gendered difference in motivational profiles, achievement, and STEM aspiration of elementary school students. Frontiers in Psychology, 13, 954325. 10.3389/fpsyg.2022.954325 36110270 PMC9469012

[41] Seetee, N. , Chi, C. , Dhir, A. , & Chen, S. (2021). Validation of the science, mathematics, and English task value scales based on longitudinal data. International Journal of Science and Mathematics Education, 19(3), 443–460. 10.1007/s10763-020-10081-x

[42] Döbert, H. (2007). Germany. In W. Hörner , H. Döbert , B. von Kopp , & W. Mitter (Eds.), The education systems of Europe (pp. 299–325). Springer.

[43] Jiang, Y. , Rosenzweig, E. Q. E. Q. , & Gaspard, H. (2018). An expectancy‐value‐cost approach in predicting adolescent students’ academic motivation and achievement. Contemporary Educational Psychology, 54, 139–152. 10.1016/j.cedpsych.2018.06.005

[44] Stoeger, H. , Duan, X. , Schirner, S. , Greindl, T. , & Ziegler, A. (2013). The effectiveness of a one‐year online mentoring program for girls in STEM. Computers and Education, 69, 408–418. 10.1016/j.compedu.2013.07.032

[45] R Core Team . (2022). R: A language and environment for statistical computing. [Computer software]. R Foundation for Statistical Computing. https://www.R‐project.org

[46] Rosseel, Y. (2012). lavaan: An R package for structural equation modeling. Journal of Statistical Software, 48(2), 1–36. 10.18637/jss.v048.i02

[47] Harpe, S. E. (2015). How to analyze Likert and other rating scale data. Currents in Pharmacy Teaching and Learning, 7(6), 836–850. 10.1016/j.cptl.2015.08.001

[48] Reichle, E. D. , Pollatsek, A. , & Rayner, K. (2006). E–Z Reader: A cognitive‐control, serial‐attention model of eye‐movement behavior during reading. Cognitive Systems Research, 7(1), 4–22. 10.1016/j.cogsys.2005.07.002

[49] Clinton‐Lisell, V. (2022). Listening ears or reading eyes: A meta‐analysis of reading and listening comprehension comparisons. Review of Educational Research, 92(4), 543–582. 10.3102/00346543211060871

[50] Ralph, B. C. W. , Thomson, D. R. , Cheyne, J. A. , & Smilek, D. (2014). Media multitasking and failures of attention in everyday life. Psychological Research, 78(5), 661–669. 10.1007/s00426-013-0523-7 24178629

[51] Kessels, U. (2014). Bridging the gap by enhancing the fit: How stereotypes about STEM clash with stereotypes about girls. International Journal of Science and Mathematics Education, 7(2), 280–296.

[52] Lewis, K. L. , Stout, J. G. , Pollock, S. J. , Finkelstein, N. D. , & Ito, T. A. (2016). Fitting in or opting out: A review of key social‐psychological factors influencing a sense of belonging for women in physics. Physical Review Physics Education Research, 12(2), 020110. 10.1103/PhysRevPhysEducRes.12.020110

[53] Statistisches Bundesamt . (2024). Niedriger Frauenanteil in technischen Studienfächern [Low percentage of women in technical fields of study] . https://www.destatis.de/Europa/DE/Thema/Bevoelkerung‐Arbeit‐Soziales/BildungKultur/Studienfachrichtungen.html

[54] Arens, A. K. (2021). Wertfacetten im Grundschulalter in drei Fächern: Differenzierung, Entwicklung, Geschlechtseffekte und Zusammenhänge zu Noten. Zeitschrift Für Pädagogische Psychologie, 35(1), 32–52. 10.1024/1010-0652/a000257

[55] Song, Y. , Guo, Y. , & Gehringer, E. F. (2017). An exploratory study of reliability of ranking vs. rating in peer assessment. International Journal of Social, Behavioral, Educational, Economic, Business and Industrial Engineering, 11, 2195–2199.

[56] Yannakakis, G. N. , & Martínez, H. P. (2015). Ratings are overrated!. Frontiers in ICT , 2. 10.3389/fict.2015.00013

[57] Vincent‐Ruz, P. , & Schunn, C. D. (2018). The nature of science identity and its role as the driver of student choices. International Journal of STEM Education, 5(1), 48. 10.1186/s40594-018-0140-5 30631738 PMC6310435

[58] Hayes, A. F. (2018). Introduction to mediation, moderation, and conditional process analysis: A regression‐based approach (2nd ed.). Guilford Press.

