# Supplementary material for: Students’ associations with the STEM acronym and their impact on value beliefs and STEM choices
Source: Ann N Y Acad Sci. 2025 Aug 25;1552(1):297–313. doi: 10.1111/nyas.70018 (PMC12576871; doi:10.1111/nyas.70018)
Supplement: Supplementary file 1 — Table S1 Demographics of the sample. Table S2 Scale evaluation of value beliefs for STEM. Table S3 Scale evaluation of academic elective intentions for STEM. Table S4 Association of subject areas with the STEM acronym. Table S5 Path models for predicting academic elective intentions for STEM by association of subject areas with the STEM acronym. Table S6 Path models for predicting STEM‐profile choices at school by the association of subject areas with the STEM acronym. [file NYAS-1552-297-s001.pdf]

Supporting Information

for Stoeger, H., Beer, A.L., & Ziegler, A.: Students' Associations with the STEM Acronym and Their Impact on Value Beliefs and STEM Choices. Annals of the New York Academy of Sciences.

Table S1

Demographics of the Sample

	Cohort 2022		Cohort 2023	
	Girls	Boys	Girls	Boys
Number of students	283	202	328	259
Mean age (range) [years]	13.7 *(12.1; 15.8)*	13.8 *(12.8; 15.0)*	13.7 *(12.7; 15.3)*	13.7 *(12.1; 15.2)*
Proportion of students with at least one parent having at least college-level education	61%	60%	60%	60%
Proportion of students born outside of Germany	15%	6%	10%	9%

Note: The table lists the demographics for both cohorts after exclusion criteria were applied.

Table S2

Scale Evaluation of Value Beliefs for STEM

No.	Item	*M*	*SD*	Factor "valence"	Factor "cost"
1	Die MINT-Fächer machen mir Spaß. [STEM subjects are fun to me.]	3.86	1.28	.660	-.300
2	Die MINT-Fächer mag ich einfach. [I simply like STEM subjects.]	3.65	1.31	.658	-.296
3	Die MINT-Fächer sind mir persönlich sehr wichtig. [STEM subjects are very important to me personally.]	3.39	1.29	.788	-.017
4	Mir ist es wichtig, in den MINT-Fächern viel zu wissen. [It is important to me to know a lot in STEM subjects.]	3.90	1.31	.809	.062
5	Die Inhalte in den MINT-Fächern werden mir in meinem Leben noch weiterhelfen. [The contents of STEM subjects will help me in my life.]	4.11	1.23	.797	.120
6	Ich werde die MINT-Fächer in meinem Leben noch oft benötigen. [I will often need STEM subjects in my life.]	3.93	1.19	.789	.191
7	Mich mit den MINT-Fächern zu beschäftigen ist anstrengend. [Dealing with STEM subjects is exhausting to me.]	3.64	1.29	.037	.794
8	Die Beschäftigung mit den MINT-Fächern kostet mich eine Menge Energie. [Dealing with STEM subjects drains a lot of my energy.]	3.40	1.29	.080	.831
9	Ich muss mich zu sehr anstrengen, um in den MINT-Fächern gut zu sein. [It takes too much of effort for me to do well in STEM subjects.]	3.28	1.32	.073	.849
10	Es kostet mich zu viel Mühe, in den MINT-Fächern eine gute Note zu bekommen. [It requires too much effort for me to get a good grade in STEM subjects.]	3.33	1.38	-.059	.742

Note: Value beliefs for STEM were examined by ten items adapted from Gaspard and colleagues[25] and Jiang and colleagues[43]. The scale question was "Wie sehr treffen die folgenden Aussagen auf dich zu?" ["How much do the following statements apply to you?"]. All items were rated on a six-point Likert scale with the following response levels: 1 (*stimmt gar nicht* [*completely disagree*]), 2 (*stimmt nicht* [*disagree*]), 3 (*stimmt eher nicht* [*rather disagree*]), 4 (*stimmt eher* [*rather agree*]), 5 (*stimmt* [*agree*]), 6 (*stimmt völlig* [*completely agree*]). A factor analysis with five factors (not shown) revealed that the factors were highly correlated around two main factors. A factor analysis with two factors (oblique promax rotation) showed that the item responses were organized along the two relatively independent factors "valence" and "cost". The table lists all items with their corresponding means (*M*), standard deviations (*SD*), and factor loadings (correlation of item responses with factor values). Note that items 1–6, which probed the intrinsic, personal importance, general utility (for future life) facets, highly loaded on the "valence" factor (with poor loading on the "cost" factor) and the items, which probed the effort required and cost facets, highly loaded on the "cost" factor (with almost no loading on the "valence" factor). The inter-factor correlation was low (r = -0.344). The same factor structure emerged when items 7–10 were reverse-coded. All questions were in German. English translations are provided in square brackets.

Table S3

Scale Evaluation of Academic Elective Intentions for STEM

No.	Item	*M*	*SD*	Item-scale correlation
1	Ich könnte mir vorstellen, ein MINT-Fach zu studieren.<br>[I can picture myself majoring in a STEM subject.]	3.36	1.51	.851
2	Ich könnte mir vorstellen, einen Beruf zu ergreifen, der etwas mit dem MINT-Bereich zu tun hat.<br>[I can picture myself pursuing a career in the STEM domain.]	3.48	1.43	.844
3	Ich könnte mir vorstellen, ein MINT-Fach als Neigungs- oder Profilfach zu wählen.<br>[I can picture myself choosing a STEM subject as an elective or focus subject.]	3.45	1.42	.835
4	Ich könnte mir vorstellen, an Angeboten wie zum Beispiel MINT-Wettbewerben oder Olympiaden teilzunehmen.<br>[I can picture myself participating in activities such as STEM competitions or Olympics.]	2.83	1.48	.751
5	Ich könnte mir vorstellen, an Angeboten wie zum Beispiel Ferienangeboten oder Nachmittagsangeboten im Schülerlabor teilzunehmen.<br>[I can picture myself participating in extracurricular STEM programs such as vacation programs or after-school lab programs.]	2.50	1.31	.731
6	Ich könnte mir vorstellen, zusätzliche MINT-bezogene schulische Angebote zu besuchen (wie z. B. AGs).<br>[I can picture myself participating in additional STEM-related school programs (e.g., clubs)]	2.75	1.38	.799

Note: Academic elective intentions for STEM were examined by six items adapted from Stoeger and colleagues[44]. The scale question was "Wie sehr treffen die folgenden Aussagen auf dich zu?" ["How much do the following statements apply to you?"]. All items were rated on a six-point Likert scale with the following response levels: 1 (*stimmt gar nicht* [*completely disagree*]), 2 (*stimmt nicht* [*disagree*]), 3 (*stimmt eher nicht* [*rather disagree*]), 4 (*stimmt eher* [*rather agree*]), 5 (*stimmt* [*agree*]), 6 (*stimmt völlig* [*completely agree*]). Although a factor analysis revealed that the items could be organized along two factors (first factor: items 1–3; second factor: items 4–6), the factors were highly correlated (inter-factor correlation r = .683). Therefore, items were combined to a one-dimensional scale. The table lists all items with their corresponding means (*M*), standard deviations (*SD*), and item-scale correlation coefficients. All questions were in German. English translations are provided in square brackets.

Table S4

Association of Subject Areas With the STEM Acronym

	Association of subject areas with the STEM acronym					
	Mathematics	Physics	Computer science	Chemistry	Biology	Engineering
Girls and boys	4.29 *(1.76)*	3.66 *(1.46)*	3.53 *(1.73)*	3.31 *(1.52)*	3.17 *(1.83)*	3.03 *(1.62)*
Girls	4.26 *(1.79)*	3.74 *(1.45)*	3.31 *(1.72)*	3.40 *(1.55)*	3.36 *(1.83)*	2.93 *(1.58)*
Boys	4.32 *(1.73)*	3.55 *(1.47)*	3.84 *(1.69)*	3.19 *(1.49)*	2.93 *(1.79)*	3.17 *(1.66)*
Difference	-.06 *($p = .59$)*	.20 *($p = .034$*)*	-.53 *($p < .001$***)*	.21 *($p = .027$*)*	.43 *($p < .001$***)*	-.24 *($p = .019$*)*

Note: The table lists the mean association of subject areas (ranks) with the STEM acronym. Association ranks were scaled from 1 (*least associated*) to 6 (*most associated*). Standard deviations (*SD*) are reported in parenthesis (italicized). Differences between girls (n = 582) and boys (n = 433) were compared by unpaired t-tests. The p values are provided in parenthesis (italicized). *) $p \leq .05$, ***) $p \leq .001$.

Table S5

Path Models for Predicting Academic Elective Intentions for STEM by Association of Subject Areas With the STEM Acronym

	Association of subject areas with the STEM acronym					
	Mathematics	Physics	Computer science	Chemistry	Biology	Engineering
	Girls and boys: mediated by cost					
Direct effect	-.003	-.008	.019	-.010	-.014	.015
	(p = .86)	*(p = .69)*	*(p = .26)*	*(p = .60)*	*(p = .40)*	*(p = .42)*
Indirect effect	-.016	.003	.006	-.006	.002	.014
	(p = .011)*	*(p = .70)*	*(p = .37)*	*(p = .41)*	*(p = .78)*	*(p = .051[a])*
	Girls and boys: mediated by valence					
Direct effect	-.019	-.015	.005	.011	-.005	.026
	(p = .11)	*(p = .28)*	*(p = .68)*	*(p = .41)*	*(p = .64)*	*(p = .045*)*
Indirect effect	-.001	.010	.020	-.028	-.007	.002
	(p = .97)	*(p = .52)*	*(p = .14)*	*(p = .072[a])*	*(p = .61)*	*(p = .87)*
	Girls: mediated by cost					
Direct effect	.031	-.023	-.017	.014	.021	-.043
	(p = .13)	*(p = .37)*	*(p = .44)*	*(p = .56)*	*(p = .29)*	*(p = .065[a])*
Indirect effect	-.011	-.016	.006	-.007	.004	.023
	(p = .30)	*(p = .24)*	*(p = .59)*	*(p = .55)*	*(p = .72)*	*(p = .067[a])*
	Girls: mediated by valence					
Direct effect	-.005	-.021	.003	.020	-.009	.012
	(p = .75)	*(p = .27)*	*(p = .83)*	*(p = .25)*	*(p = .56)*	*(p = .49)*
Indirect effect	.025	-.018	-.014	-.014	.034	-.032
	(p = .16)	*(p = .41)*	*(p = .44)*	*(p = .50)*	*(p = .047*)*	*(p = .11)*
	Boys: mediated by cost					
Direct effect	-.062	.025	.077	-.049	-.070	.087
	(p = .022)*	*(p = .43)*	*(p = .005**)*	*(p = .12)*	*(p = .007**)*	*(p = .002**)*
Indirect effect	-.016	.017	-.002	-.001	.005	.003
	(p = .015)*	*(p = .031*)*	*(p = .76)*	*(p = .91)*	*(p = .41)*	*(p = .67)*
	Boys: mediated by valence					
Direct effect	-.042	-.010	.021	-.008	-.012	.052
	(p = .026)*	*(p = .64)*	*(p = .29)*	*(p = .72)*	*(p = .51)*	*(p = .007**)*
Indirect effect	-.036	.052	.055	-.041	-.053	.037
	(p = .071[a])	*(p = .029*)*	*(p = .008**)*	*(p = .079[a])*	*(p = .006**)*	*(p = .078[a])*

Note. The table lists the path coefficients (*b*) of the mediation analyses that tested whether the association of subject areas with the STEM acronym predicted academic elective intention for STEM either directly or mediated (indirectly) via the two subscales of value beliefs for STEM (valence and cost). The models were examined for the combined group (girls and boys) and separately for girls (n = 582) and boys (n = 433). Path coefficients for direct and indirect effects were tested by Zobel *Z*-tests. The *p* values are provided in parentheses. [a]) $p \leq .1$, *) $p \leq .05$, **) $p \leq .01$.

Table S6

Path Models for Predicting STEM-Profile Choices at School by the Association of Subject Areas With the STEM Acronym

	Association of subject areas with the STEM acronym					
	Mathematics	Physics	Computer science	Chemistry	Biology	Engineering
			Girls and boys: mediated by cost			
Direct effect	-.042	-.040	.057	-.090	-.020	.127
	(p = .27)	*(p = .30)*	*(p = .13)*	*(p = .020*)*	*(p = .61)*	*(p < .001***)*
Indirect effect	-.028	.004	.010	-.009	.003	.021
	(p = .014)*	*(p = .69)*	*(p = .40)*	*(p = .41)*	*(p = .78)*	*(p = .055[a])*
			Girls and boys: mediated by valence			
Direct effect	-.069	-.047	.043	-.069	-.008	.145
	(p = .055[a])	*(p = .22)*	*(p = .23)*	*(p = .058[a])*	*(p = .82)*	*(p < .001***)*
Indirect effect	-.001	.011	.024	-.030	-.008	.003
	(p = .97)	*(p = .51)*	*(p = .14)*	*(p = .071[a])*	*(p = .62)*	*(p = .87)*
			Girls: mediated by cost			
Direct effect	.022	-.044	-.054	-.073	.067	.067
	(p = .67)	*(p = .41)*	*(p = .29)*	*(p = .15)*	*(p = .19)*	*(p = .18)*
Indirect effect	-.016	-.018	.008	-.009	.006	.029
	(p = .33)	*(p = .26)*	*(p = .61)*	*(p = .56)*	*(p = .71)*	*(p = .068[a])*
			Girls: mediated by valence			
Direct effect	-.027	-.043	-.027	-.066	.027	.134
	(p = .56)	*(p = .40)*	*(p = .56)*	*(p = .17)*	*(p = .59)*	*(p = .004**)*
Indirect effect	.033	-.019	-.018	-.016	.046	-.039
	(p = .17)	*(p = .42)*	*(p = .45)*	*(p = .49)*	*(p = .050*)*	*(p = .12)*
			Boys: mediated by cost			
Direct effect	-.147	-.002	.139	-.088	-.081	.176
	(p = .013)*	*(p = .97)*	*(p = .020*)*	*(p = .14)*	*(p = .18)*	*(p = .003**)*
Indirect effect	-.036	.032	-.004	-.002	.011	.006
	(p = .012)*	*(p = .020*)*	*(p = .78)*	*(p = .91)*	*(p = .43)*	*(p = .68)*
			Boys: mediated by valence			
Direct effect	-.141	-.021	.073	-.048	-.006	.141
	(p = .015)*	*(p = .71)*	*(p = .20)*	*(p = .41)*	*(p = .91)*	*(p = .013*)*
Indirect effect	-.042	.051	.061	-.041	-.064	.041
	(p = .087[a])	*(p = .030*)*	*(p = .011*)*	*(p = .094[a])*	*(p = .016*)*	*(p = .077[a])*

Note. The table lists the path coefficients (b) of the mediation analyses that tested whether the association of subject areas with the acronym STEM predicted the STEM-profile choices at school (STEM vs. non-STEM) for the upcoming school year either directly or mediated (indirectly) via the two subscales of value beliefs (valence and cost). The models were examined for the combined group (girls and boys) and separately for girls (n = 582) and boys (n = 433). Path coefficients for direct and indirect effects were tested by Zobel Z-tests. The p values are provided in parentheses. ^{a}) $p \leq .1$, *) $p \leq .05$, **) $p \leq .01$, ***) $p \leq .001$.
